# SLAM-ITseq: sequencing cell type-specific transcriptomes without cell sorting

**DOI:** 10.1242/dev.164640

**Published:** 2018-07-11

**Authors:** Wayo Matsushima, Veronika A. Herzog, Tobias Neumann, Katharina Gapp, Johannes Zuber, Stefan L. Ameres, Eric A. Miska

**Affiliations:** 1 Department of Genetics, University of Cambridge, Downing Street, Cambridge CB2 3EH, UK; 2 Wellcome Trust Cancer Research UK Gurdon Institute, University of Cambridge, Tennis Court Road, Cambridge CB2 1QN, UK; 3 Wellcome Trust Sanger Institute, Wellcome Trust Genome Campus, Cambridge CB10 1SA, UK; 4 Institute of Molecular Biotechnology, Vienna Biocenter Campus, Vienna 1030, Austria; 5 Research Institute of Molecular Pathology, Vienna Biocenter Campus, Vienna 1030, Austria

**Keywords:** RNA-seq, RNA *in vivo* labelling, 4-thiouracil, Transcriptomics, Transgenics

## Abstract

Cell type-specific transcriptome analysis is an essential tool for understanding biological processes in which diverse types of cells are involved. Although cell isolation methods such as fluorescence-activated cell sorting (FACS) in combination with transcriptome analysis have widely been used so far, their time-consuming and harsh procedures limit their applications. Here, we report a novel *in vivo* metabolic RNA sequencing method, SLAM-ITseq, which metabolically labels RNA with 4-thiouracil in a specific cell type *in vivo* followed by detection through an RNA-seq-based method that specifically distinguishes the thiolated uridine by base conversion. This method has successfully identified the cell type-specific transcriptome in three different tissues: endothelial cells in brain, epithelial cells in intestine and adipocytes in white adipose tissue. As this method does not require isolation of cells or RNA prior to the transcriptomic analysis, SLAM-ITseq provides an easy yet accurate snapshot of the transcriptional state *in vivo*.

## Abstract (precis)

**Highlighted Article:** A novel *in vivo* metabolic RNA sequencing method, SLAM-ITseq, enables cell type-specific transcriptome analysis without time-intensive cell or RNA sorting steps, making it accessible to a broader research area.

## INTRODUCTION

Animals consist of various organs, which are further composed of heterogeneous populations of highly specialised cells. Thus, it is important to look at transcriptomic changes at the cellular level to understand animal physiology. To capture the transcriptome of a specific cell type, mechanical cell isolation methods such as fluorescence-activated cell sorting (FACS) or laser-capture microdissection (LCM) prior to RNA quantification have widely been used so far. Moreover, combined with such cell-isolation methods, the recent advance in high-throughput RNA sequencing (RNA-seq) methods now enables us to quantitate transcripts at single-cell resolution (Tang et al., 2010). However, as the transcriptome of a cell is greatly affected by its cellular context as well as mechanical/chemical stimuli, it has been questioned how closely transcriptomic data obtained from sorted cells reflect the state prior to cell sorting (Richardson et al., 2015). In addition, these cell-isolation methods are often time-intensive, involve laborious steps and lead to considerable cell death after isolation, which limits their applications to robust cells only.

Recently, Gay et al. developed an elegant *in vivo* metabolic RNA-labelling method, TU-tagging, to study cell type-specific transcriptomes, using a uracil analogue 4-thiouracil (Miller et al., 2009; Gay et al., 2013). *Toxoplasma gondii* (*T. gondii*) uracil phosphoribosyltransferase (UPRT) converts 4-thiouracil to 4-thiouridine monophosphate (4-thio-UMP), which is incorporated into newly synthesised RNA molecules to generate thiol-containing RNA (thio-RNA). As UPRT homologs in many organisms, including mice, are enzymatically inactive (Cleary et al., 2005), cells in these animals cannot use 4-thiouracil to synthesise thio-RNA. Gay et al. generated transgenic mice expressing *T. gondii* UPRT (herein, UPRT refers to *T. gondii* UPRT unless otherwise stated) in a specific cell type and exposed them to 4-thiouracil to ‘label’ newly synthesised RNA. Then, they pulled-down the thio-RNA using a biochemical isolation method and quantified by RNA-seq to determine enrichment level of labelled RNA over the total RNA. Even though similar methods have been tested in various model organisms (Erickson and Nicolson, 2015; Chatzi et al., 2016; Tomorsky et al., 2017), technical and analytical challenges limit their application. First, the biochemical isolation methods of thio-RNA have been shown to have high background noise, which makes it difficult to distinguish lowly labelled RNA from the background noise. This issue is especially pronounced when used *in vivo*, where relatively low 4-thiouracil concentrations can be achieved. Second, because the pulled-down RNA and input RNA are sequenced separately, unbiased estimation of the labelling level of a given transcript is difficult unless well-designed spike-in is included. In addition, labelling level estimation used in TU-tagging is not optimal when studying tissues where UPRT-expressing cells are the dominant cell type. Identification of labelled transcripts relies on the enrichment of pulled-down read counts over input read counts, which is very similar in the above condition (see discussion in Gay et al., 2013).

Here, we describe a new method that significantly improves *in vivo* metabolic labelling (Fig. S1): we redesigned the experiment to use a different control to account for background labelling, used the RNA-seq method called thiol(SH)-linked alkylation for the metabolic sequencing of RNA (SLAMseq) to directly identify thiol-containing uracil at single-base resolution (Herzog et al., 2017a), and applied a statistical method to reliably identify labelled transcripts, accounting for biological variance in the labelling level. This improved method, which we now term SLAMseq in tissue (SLAM-ITseq), makes the *in vivo* 4-thiouracil-based metabolic labelling methods accessible to wider research areas to study cell type-specific transcriptomics in animals.

## RESULTS AND DISCUSSION

### Experimental design of SLAM-ITseq

**Fig. 1.**
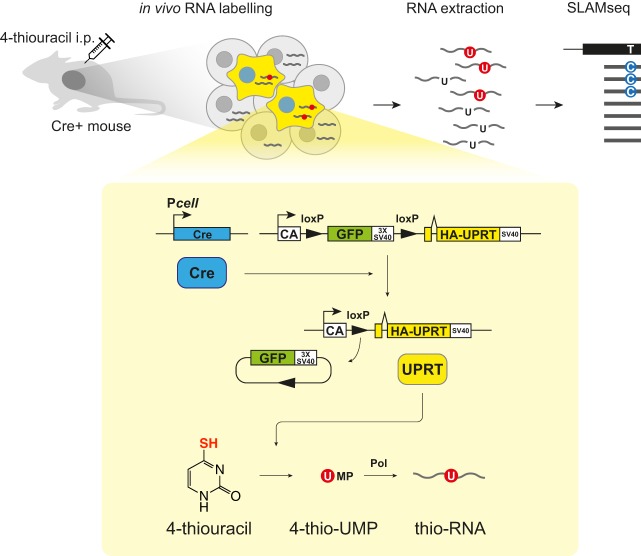
**SLAM-ITseq design.** Schematic of how SLAM-ITseq works. Cre is expressed in cells in which a cell type-specific promoter (P*cell*) is activated and removes the floxed GFP-coding sequence followed by simian virus 40 polyadenylation sequence (SV40) between chicken β-actin promoter (CA) and UPRT-coding region, resulting in UPRT expression in the cells. When the mice are exposed to 4-thiouracil, only those UPRT-expressing cells (shown in yellow) can convert 4-thiouracil to 4-thio-UMP to synthesise thio-RNA. RNA is extracted from entire tissue without cell sorting, and the labelled RNA that is synthesised in the cells of interest is identified by finding T>C containing reads using SLAMseq.

To generate mice expressing UPRT in a cell type of interest, we crossed mice carrying Cre recombinase (Cre) under a cell type-specific promoter (*Cre* mice) with previously developed *UPRT* transgenic mice, which express haemagglutinin(HA)-tagged UPRT in a Cre-inducible manner. From a cross of homozygous *UPRT* mice (*uprt/uprt*) and hemizygous *Cre* mice (*cre/0*), both *uprt/0; cre/0* (Cre^+^) and *uprt/0; +/+* (Cre^−^) mice were obtained. When Cre^+^ mice are exposed to 4-thiouracil, the RNA synthesised in the cells expressing UPRT is labelled. To identify the labelled transcripts, RNA extracted from the whole tissue was treated with iodoacetamide (IAA) to alkylate the thiol group of the thio-RNA and then subsequently used as RNA-seq input. During the reverse transcription step of RNA-seq library preparation, a guanine (G), instead of an adenine (A), is base-paired to an alkylated 4-thiouracil leading to the thymine to cytosine base conversion (T>C) at the corresponding T position in the reads generated from the thio-RNA. T>C mismatch-aware alignment and T>C counting per gene were performed (Fig. 1). To control for the background labelling and to capture both specific and common transcripts of a certain cell type, RNA obtained from Cre^−^ mice that were subject to the same procedures was also prepared.

### SLAM-ITseq identified endothelial transcripts sensitively and specifically

**Fig. 2.**
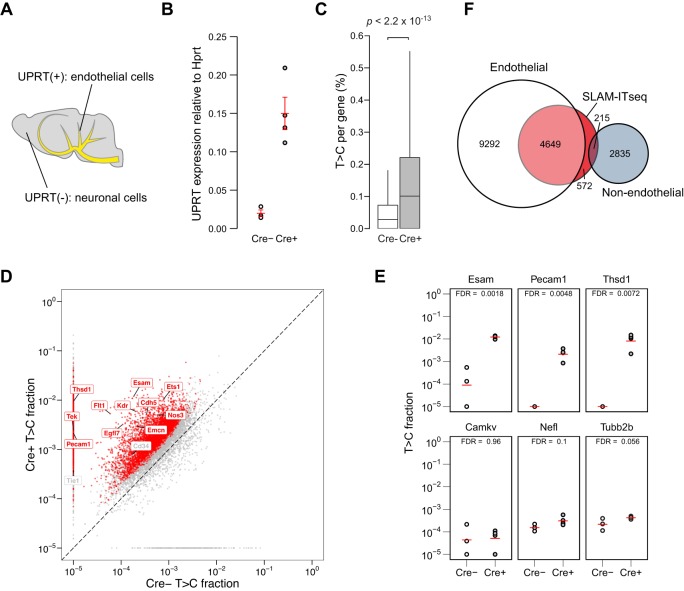
**Analyses of labelled RNA from the mouse brain expressing UPRT in endothelial cells.** (A) Schematic of UPRT-expressing cells (yellow) and non-UPRT-expressing cells (grey) in the Cre^+^ mouse brain. (B) Comparison of UPRT mRNA expression by RT-qPCR in total brain RNA from Cre^+^ or Cre^−^ animals. The red bars indicate the mean expression and 95% confidence intervals among biological replicates (Cre^+^: *n*=4, Cre^−^: *n*=3). (C) Comparison of T>C rate in all T positions sequenced per gene. Data are shown as boxplots. The lower and upper hinges correspond to the first and third quartiles, the middle hinges indicate the median and the whiskers extend to 1.5 interquartile range from the upper hinges. Outliers are omitted from the plot. Two-tailed Mann–Whitney *U*-test was used to calculate the *P*-value. (D) Mean T>C fractions of each gene are plotted. Labels indicate the genes that are known to be expressed in endothelial cells. Significantly more labelled transcripts in Cre^+^ were determined by beta-binomial test and shown as red points and label (FDR<0.05). A constant value of 1×10^−5^ was added to the raw T>C value when plotting. (E) T>C fraction of known endothelial cell-specific genes (*Esam*, *Pecam1* and *Thsd1*) and neuronal cell-specific genes (*Camkv*, *Nefl* and *Tubb2b*) are shown. The red bars indicate the mean T>C fraction of biological replicates (Cre^+^: *n*=4, Cre^−^: *n*=3). (F) Euler diagram comparing labelled genes identified using SLAM-ITseq, and endothelial and non-endothelial genes identified with a FACS experiment.

First, to compare SLAM-ITseq to the previously described TU-tagging, we generated the same double-transgenic mice used in the TU-tagging experiment, which label RNA in endothelial cells specifically by crossing *Tie2-Cre* mice and *UPRT* mice (Kisanuki et al., 2001; Gay et al., 2013) (Fig. 2A).

After exposing both Cre^+^ and Cre^−^ animals to 4-thiouracil for 4 h, RNA was extracted from the whole brain of each animal. To confirm controlled *UPRT* transgene expression, reverse transcription followed by quantitative polymerase chain reaction (RT-qPCR) was performed on complementary DNA (cDNA) obtained from each animal. As expected, we confirmed that UPRT was expressed only in Cre^+^ animals (Fig. 2B), and significantly more T>Cs were observed in Cre^+^ animals (Fig. 2C). Importantly, the read count for each gene remained unchanged between Cre^+^ and Cre^−^ animals (Fig. S2A), suggesting that neither Cre nor UPRT expression has considerable effect on the transcriptome itself.

Next, we employed a statistical approach to identify the labelled transcripts. As nucleotide conversion is a binomial process and the probability of it occurring at a given T position can be modelled by beta distribution for biological replicates, beta-binomial distribution can describe the T>C fraction per gene among biological replicates. Based on the variance estimate of T>C fraction (Table S1), genes that are significantly labelled in Cre^+^ were identified at FDR<0.05 (Fig. 2D). To evaluate the effect of T content per gene on the significance calling, proportions of labelled transcripts in different T content were compared (Fig. S2B). Although slightly higher proportion of labelled genes were observed in the bins of higher T content, considering there are labelled genes with fewer than 50 T bases and those genes represent minor fraction of the population, it suggests that the paucity of T bases in a gene has little effect on the discovery of labelled transcripts.

To evaluate the sensitivity of this significance calling, we used the same set of known endothelial genes selected and tested for TU tagging as a positive control. As expected, out of 13 known endothelial genes (*Cdh5*, *Cd34*, *Egfl7*, *Emcn*, *Esam*, *Ets1*, *Flt1*, *Kdr*, *Nos3*, *Pecam1*, *Tek*, *Tie1* and *Thsd1*), 11 were called as significant, all apart from *Tie1* and *Cd34* (Fig. 2D). As *Tie1* and *Cd34* were not significantly labelled by TU-tagging, these genes might not be actively transcribed. T>C fractions of some known endothelial and neuronal marker genes are shown (Fig. 2E). Interestingly, some known housekeeping genes, such as *Hprt* and *Actb*, are also significantly labelled (Fig. S2C). As these genes should be expressed globally in brain, it is suggestive that SLAM-ITseq is sensitive enough to detect small shift in the labelled fraction despite the majority of a given gene being unlabelled. Next, to compare SLAM-ITseq with the conventional FACS-based method, we used a published RNA-seq dataset from multiple cell types that were sorted from mouse cortices by FACS for transcriptome analysis (Zhang et al., 2014). Importantly, transgenic GFP controlled under the same endothelial-specific promoter (*Tie2-GFP*) was used to isolate endothelial cells in this study. Based on the FPKM value in the dataset, we have defined the genes that are expressed in endothelial cells, and genes that are expressed only in non-endothelial cells in the brain (see Materials and Methods for further details). As shown in the Euler diagram (Fig. 2F), although more than 85% of labelled genes in SLAM-ITseq were also detected in the FACS-isolated endothelial cells, fewer than 4% of those were included as non-endothelial genes, i.e. potential false positives. It is reasonable that the number of labelled genes in SLAM-ITseq is fewer than the number of genes detected by the FACS method, as the labelling time by 4-thiouracil was 4 h, and thus only transcripts that were actively synthesised within this time window should be labelled.

To comprehensively analyse the functional profile of significantly labelled genes, gene ontology (GO) enrichment analysis was performed on the labelled gene list. The biological process GO terms in the list were enriched for a cluster represented by ‘cardiovascular system development’, which is known to be related to the endothelial functions in addition to all the other general terms (Fig. S2D). Together, these results indicate the successful labelling of endothelial transcripts without labelling the transcripts synthesised in surrounding cells.

### A cell type-specific transcriptome was identified in different murine tissues

**Fig. 3.**
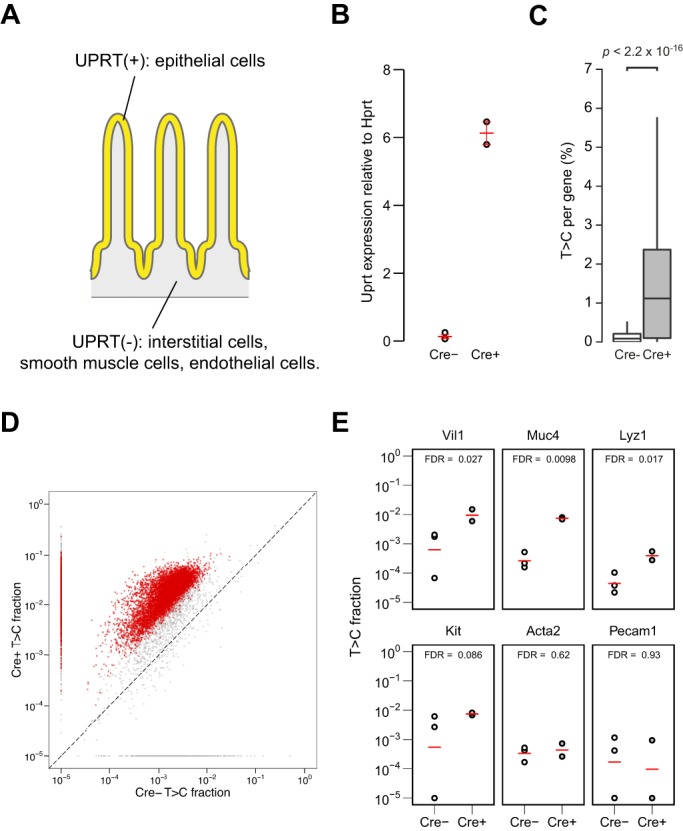
**SLAM-ITseq analyses of labelled RNA from the mouse duodenum expressing UPRT in epithelial cells.** (A) Schematic of UPRT-expressing cells (yellow) and non-UPRT-expressing cells (grey) in the Cre^+^ mouse intestine. (B) Comparison of UPRT mRNA expression by RT-qPCR in total duodenum RNA from the Cre^+^ and Cre^−^ animals. The red bars indicate the mean expression and 95% confidence intervals (Cre^+^: *n*=2, Cre^−^: *n*=3). (C) Comparison of T>C rate in all T positions sequenced per gene. Data are shown as boxplots. The lower and upper hinges correspond to the first and third quartiles, the middle hinges indicate the median, and the whiskers extend to 1.5 interquartile range from the upper hinges. Outliers are not shown. Two-tailed Mann–Whitney *U*-test was used to calculate the *P*-value indicated. (D) Mean T>C fractions of each gene are plotted. Significantly more labelled transcripts in Cre^+^ were determined using a beta-binomial test and shown as red points (FDR<0.05). A constant value of 1×10^−5^ was added to the raw T>C value when plotting. (E) T>C fraction of known intestinal epithelium-specific genes (*Vil1*, *Muc4* and *Lyz1*) and genes known to be transcribed in non-epithelial cells in small intestine: *Kit*, an interstitial gene; *Acta2*, a smooth muscle gene; *Pecam1*, an endothelial gene. The red bars indicate the mean T>C fraction of biological replicates (Cre^+^: *n*=2, Cre^−^: *n*=3).

**Fig. 4.**
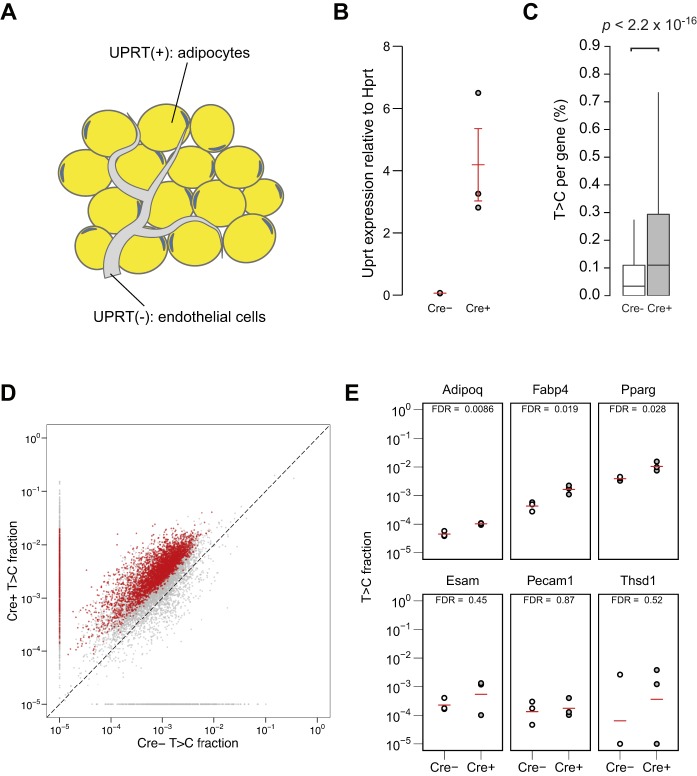
**SLAM-ITseq analyses of labelled RNA from the mouse eWAT expressing UPRT in adipocytes.** (A) Schematic of UPRT-expressing cells (yellow) and non-UPRT-expressing cells (grey) in the Cre^+^ mouse eWAT. (B) Comparison of UPRT mRNA expression by RT-qPCR in eWAT from the Cre^+^ and Cre^−^ animals. The red bars indicate the mean expression and 95% confidence intervals among biological replicates. (C) Comparison of T>C rate in all T positions sequenced per gene. Data are shown as boxplots. The lower and upper hinges correspond to the first and third quartiles, the middle hinges indicate the median, and the whiskers extend to 1.5 interquartile range from the upper hinges. Outliers are not shown. The *P*-value obtained by two-tailed Mann–Whitney *U*-test is shown. (D) Mean T>C fractions of each gene are plotted. Significantly more labelled transcripts in Cre^+^ were determined by beta-binomial test and shown as red points (FDR<0.05). A constant value of 1×10^−5^ was added to the raw T>C value when plotting. (E) T>C fraction of known adipocyte-specific genes (*Adipoq*, *Fabp4* and *Pparg*) and genes known to be transcribed in endothelial cells (*Esam*, *Pecam1* and *Thsd1*). The red bars indicate the mean T>C fraction of biological replicates (Cre+: *n*=3, Cre^−^: *n*=3).

Next, we examined whether this method is applicable to study transcriptomes of other cell types, using different promoters for the Cre expression control. *Vil-Cre* (Madison et al., 2002) and *Adipoq-Cre* (Eguchi et al., 2011) mice were crossed with the *UPRT* mice to generate mice that specifically express UPRT in adipocytes and gut epithelial cells, respectively. It is important to note that the ratio of Vil^+^ cells in intestine and Adipoq^+^ cells in adipose tissue are much higher than Tie2^+^ cells in the brain (Figs 3A and 4A), and thus transcriptomic analysis of these cell types had been difficult with TU-tagging. RNA was extracted from epididymal white adipose tissue (eWAT) and duodenum from each set of mice. Cre-dependent UPRT expression was confirmed by RT-qPCR (Figs 3B and 4B). To identify the labelled RNA, SLAMseq was performed on the RNA extracted from each tissue. Beta-binomial test comparing Cre^+^ and Cre^−^ as in the previous analysis identified significantly labelled genes in Cre^+^ (Figs 3D and 4D). The genes known to be expressed in the Cre-expressing cells were significantly labelled, whereas those known to be expressed in the other cell types in the tissue were not labelled (Figs 3E and 4E and Tables S2 and S3). For comprehensive analysis of labelled genes in each strain, GO term enrichment analysis was performed (Figs S3 and S4). Although *Adipoq-Cre^+^* cells revealed to enrich for GO terms represented by ‘fat cell differentiation’ (Fig. S4), which is related to adipocytes, no such specific cluster was observed in *Vil-Cre^+^* results (Fig. S3). This could be because the *Vil-Cre^+^* cells consist of heterogeneous types of cells, which have distinct roles, and thus did not enrich for specific terms as a whole. These results demonstrate that SLAM-ITseq can identify cell type-specific transcripts using Cre lines specific to a wide range of tissues, regardless of the proportion of the cell type of interest in a tissue.

### Conclusions

SLAM-ITseq dramatically broadens the application of SLAMseq to the study of cell type-specific transcriptomes *in vivo* in a wide range of tissues by circumventing laborious and potentially disturbing cell isolation steps. The results above show that this method is applicable to different murine tissues by simply using an existing cell type-specific Cre line. Its sensitivity and specificity were confirmed by examining successful labelling of genes that are known to be specific for each cell type in the tissues examined. Extended exposure time could potentially increase the labelling level and the number of labelled transcripts, which could be of benefit when the steady-state transcriptome is sought.

Since TU-tagging was reported, there have been several other papers describing cell type-specific transcriptome analysis independent of cell isolation. One potential problem with the use of 4-thiouracil for tagging is that it could potentially be incorporated to RNA independent of external UPRT expression either by negligible endogenous Uprt activity or by an unknown alternative salvage pathway. To overcome this problem, 5-ethynylcytosine (EC) was suggested as an alternative to 4-thiouracil, because the incorporation of EC to RNA requires cytosine deaminase (CD) in addition to UPRT, and thus might increase the specificity (Hida et al., 2017). However, this method requires an antibody-based procedure to isolate EC-tagged RNA, which could add another layer of background noise due to nonspecific binding of unlabelled RNA to the antibody. As our method overcame the background labelling problem by using control animals exposed to 4-thiouracil and does not involve an RNA-isolation step, a direct comparison might be needed to determine whether this method has a higher sensitivity than SLAM-ITseq. Another approach involves the expression of a tagged RNA-binding protein in a specific cell type and the pulling-down of the tagged protein to co-purify the RNA bound to it. By using different RNA-binding proteins, one can study various RNA profiles: tagged ribosomal protein enables the study of the ‘translatome’ by identifying RNA bound to ribosomes (Hupe et al., 2014), while tagged polyA-binding protein (PABP) identifies alternative polyadenylation (APA) events in a cell-specific manner (Hwang et al., 2017). To study small RNA transcriptome, transgenic cell-type specific expression of a plant-specific methyltransferase, HEN1, which introduces 3′-terminal 2′-O-methylation, has been used and the methylated miRNA was identified by using a methylation-dependent small RNA-seq method (Alberti et al., 2018). As Alberti et al. did not apply this method in mice, it would be interesting to compare to SLAM-ITseq, with which we also detected labelled miRNA when small RNA-seq was performed (data not shown).

The potential applications of SLAM-ITseq go well beyond the identification of a cell type-specific transcriptome. As it exclusively labels transcripts that are synthesised while cells are exposed to 4-thiouracil, it might better capture dynamic transcriptional change when combined with any perturbation given to the animal. In addition, it could potentially differentiate the direct transcriptional change induced by a perturbation from secondary effects, in a similar manner as SLAMseq successfully identifies direct targets of several transcription factors *in vitro* (Muhar et al., 2018). Thus, SLAM-ITseq provides unparalleled access to cellular transcriptional dynamics to better understand animal physiology.

## MATERIALS AND METHODS

### Animal husbandry

All mice were maintained in a specific pathogen-free facility with sentinel monitoring at standard temperature (19-23°C) and humidity (55±10%), on a 12 h dark/12 h light cycle (lights on 0730–1900) and fed standard rodent chow (LabDiet 5021-3, 9% crude fat content, 21% kcal as fat, 0.276 ppm cholesterol). Both food and water were available *ad libitum*. The mice were housed in groups of three or four per cage in individually ventilated caging receiving 60 air changes per hour. In addition to Aspen bedding substrate, standard environmental enrichment of a nestlet and a cardboard tunnel were provided. All animals were regularly monitored for health and welfare concerns, and were additionally checked prior to and after procedures. The care and use of mice in the study was carried out in accordance with UK Home Office regulations, UK Animals (Scientific Procedures) Act of 1986 under a UK Home Office license that approved this work (PF8733E07), which was reviewed regularly by the Wellcome Sanger Institute Animal Welfare and Ethical Review Body.

### Generation of transgenic mice

All mice used were crossed with C57BL/6NTac at least once before used for further crossings. Homozygous *UPRT* transgenic mice (Gay et al., 2013) (*uprt/uprt*) were crossed with hemizygous *Cre* mice (*Tie2-Cre*, JAX stock #008863; *Vil-Cre*, JAX stock #004586; and *Adipoq-Cre*, JAX stock #010803) (Kisanuki et al., 2001; Madison et al., 2002; Eguchi et al., 2011) (*cre/0*), and then *uprt/0; cre/0* (Cre^+^) and *uprt/0; +/+* (Cre^−^) animals were obtained as resulting offspring. To confirm the genotype, DNA from mouse ear-clips was isolated using the Sample-to-SNP kit (Life Technologies), and products amplified using a Viia7 qPCR machine (Life Technologies). Results were analysed against known calibrator controls using the ddCt method (Livak and Schmittgen, 2001). TaqMan (Life Technologies) qPCR assay sequences against UPRT used the following primers: forward, 5′-ATTCCAAGATCTGTGGCGTC-3′; reverse, 5′-CTTCTCGTAGATCAGCTTAGGC-3′; probe (VIC), 5′-CCGCATCGGGAAAATCCTCATCCA-3′. Primers against Cre were: forward, 5′-ACGTACTGACGGTGGGAGAA-3′; reverse, 5′-GTGCTAACCAGCGTTTTCGTT-3′; probe (VIC), 5′-CTGCCAATATGGATTAACA-3′.

### 4-Thiouracil administration

4-Thiouracil administration was performed using the previously reported methods (Gay et al., 2013, 2014). Briefly, 4-thiouracil was dissolved in DMSO at 200 mg/ml concentration followed by further dilution in corn oil (1:4). This solution (8 µl/g body weight; i.e. 400 mg/kg body weight of 4-thiouracil) was intraperitoneally injected to 8-10 week old both male and female Cre^+^ and Cre^−^ mice using a 25 G×⅝” needle (Terumo). Number of animals used (>2) was determined based on the minimum number required for the statistical test used (beta-binomial test). Mice were culled and tissues harvested 4 h after the injection. The collected tissues were cut into small pieces (less than 5 mm in thickness) and submerged in RNAlater (Sigma-Aldrich) for storage at −20°C.

### RNA extraction from tissues

After removing RNAlater using clean Kimtech (Kimberly-Clark), around 30 mg of tissue was homogenised in 1 ml TRIsure (Bioline), using TissueLyser LT (Qiagen) and 7 mm stainless steel beads (Qiagen). RNA extraction was performed following the manufacturer's instruction with minor modifications: the isopropanol precipitation step was carried out with the addition of 20 µg/ml glycogen and 0.1 µM DTT and the isopropanol precipitation at −20°C was extended to 2 h. Purified RNA was subsequently treated with 1 U of TURBO DNase (Invitrogen) to eliminate potential DNA contamination. After the DNase reaction, RNA was cleaned up using RNA Clean & Concentrator-5 (Zymo Research) and stored at −80°C with 1 mM DTT in order to prevent oxidation.

### RT-qPCR

Reverse transcription was performed using SuperScript II Reverse Transcriptase (Invitrogen) and random hexamers (Invitrogen) following the manufacturer's instruction. Around 10 µg of the synthesised cDNA was used as an input for 10 µl qPCR reaction using PowerUp SYBR Green Master Mix (Applied Biosystems) with the gene-specific primer pairs targeting *T. gondii* UPRT (forward, 5′-CCCGATATTCGACAAACGAC-3′; reverse, 5′-GCTTCATGAGCACCACATTG-3′) and *M. musculus* Hprt (forward, 5′-GCCTAAGATGAGCGCAAGTTG-3′; reverse, 5′-TACTAGGCAGATGGCCACAGG-3′). Technical triplicates were prepared and the mean CT value was calculated for each biological replicate.

### SLAMseq

DNase-treated RNA was reacted with IAA to alkylate the thiol group following the protocol previously described (Herzog et al., 2017b). Briefly, 50 µl reaction mix [5-10 µg RNA, 10 mM IAA, 50 mM (pH 8) sodium phosphate, and 50% DMSO] was incubated at 50°C for 15 min. The reaction was stopped by adding 1 µl of 1 M DTT, followed by 1 µl glycogen (20 mg/ml), 5 µl NaOAc (3 M, pH 5.2) and 125 µl 100% ethanol. After 2 h incubation at −20°C, the solution was centrifuged and the obtained RNA pellet was washed with 80% ethanol. The pellet was resuspended in 15 μl nuclease-free water. RNA concentration was quantified using Qubit RNA BR Assay Kit (Molecular Probes) and 500 ng of it was used as an input for QuantSeq 3′ mRNA-Seq Library Prep Kit for Illumina (Lexogen). RNA-seq library preparation was conducted following manufacturer's instructions with the PCR cycles optimised for different tissues (brain, 13; duodenum, 15; eWAT, 15). The multiplexed libraries were sequenced using HiSeq 1500 (Illumina) for single-end 100 cycles by the core NGS service at the Gurdon Institute, Cambridge, UK.

### Bioinformatic pipeline for labelled genes identification

Demultiplexed fastq files were first analysed with FastQC (version 0.11.5) for quality check. These sequence data were then analysed by the software designed for SLAMseq analysis, SLAM-DUNK (version 0.2.4, t-neumann.github.io/slamdunk/), to quantify how many T>Cs are detected per gene in each sample. For the mapping and SNP calling, *Mus musculus* primary genome assembly GRCm38 was used. For counting T>C per gene, 3′UTR annotation data from Refseq and Ensembl was used. The other parameters used were as follows: −5 12 −n 100 −m −mv 0.2 −mts −rl 100.

To identify significantly labelled transcripts, first, genes that had zero coverage on T in any sample were excluded. Next, the number of T>Cs and the total T coverage of the genomic sequence in each annotated gene were used to perform two-sided beta-binomial test by the R package ibb (version 13.06) (Pham et al., 2010). To control the false discovery rate (FDR), the Benjamini-Hochberg procedure was employed on the calculated *P*-value, and FDR <0.05 was set to determine the significantly labelled transcripts.

### FACS dataset analysis

A published data table containing FPKM value for each gene in multiple cell types isolated from brain has been further analysed (Zhang et al., 2014). Zhang et al. reasoned the use of FPKM >0.1 as a conservative threshold to determine significantly expressed genes (>99% confidence) and rounded up FPKM values <0.1 to 0.1 to avoid inflated values when calculating ratios. Thus, the genes detected in the sorted endothelial cells were determined by filtering genes that have FPKM >0.1 in endothelial cells; the genes expressed in only non-endothelial cells were identified by choosing the genes with mean FPKM >0.1 among non-endothelial cells and FPKM=0.1 in endothelial cells.

### GO term enrichment analysis

The list of all the annotated genes was sorted by *P*-value from beta-binomial test in ascending order and then used as input for PANTHER 13.1 (pantherdb.org/) (Mi et al., 2017) to perform ‘Statistical enrichment test’ with full biological processes GO terms. The enriched GO terms obtained were further analysed using REVIGO (revigo.irb.hr/) (Supek et al., 2011) with allowed similarity=0.4 to better visualise the results with less redundancy of GO terms.

## Supplementary Material

Supplementary information
