# Supplementary material for: SLAM-ITseq: sequencing cell type-specific transcriptomes without cell sorting
Source: Development. 2018 Jul 11;145(13):dev164640. doi: 10.1242/dev.164640 (PMC6053661; doi:10.1242/dev.164640)
Supplement: Supplementary information [file develop-145-164640-s1.pdf]

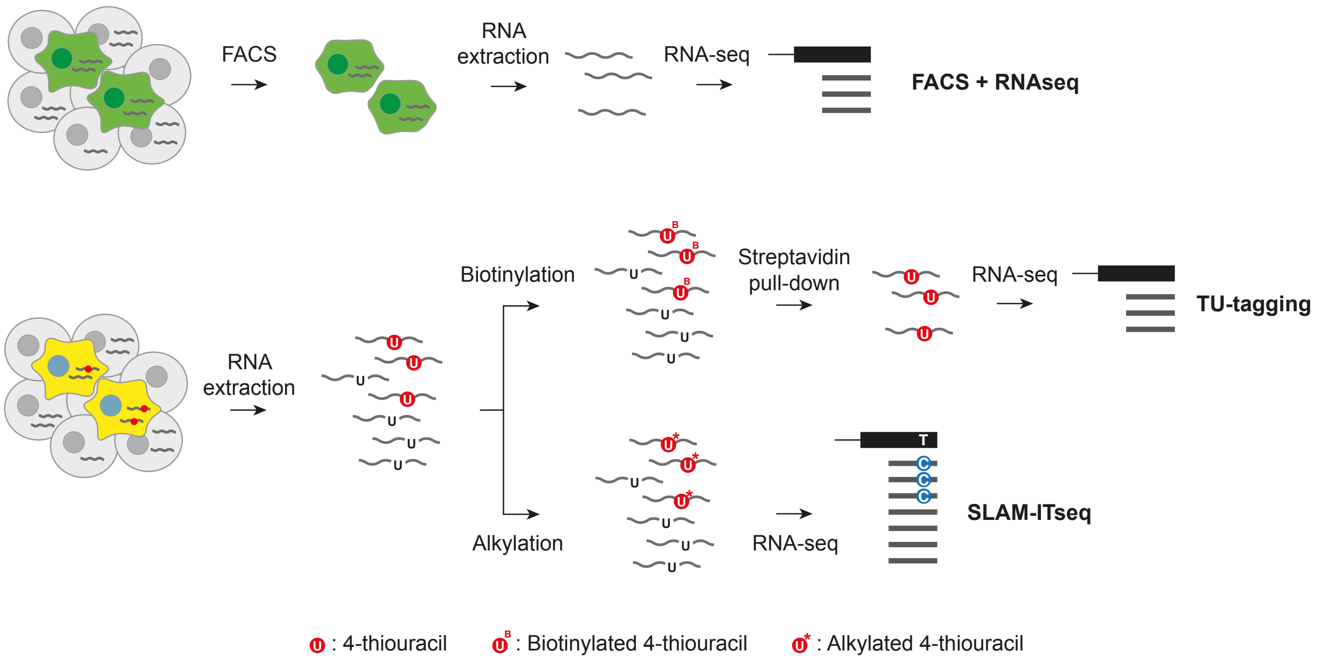

Figure S1. Comparison of different cell type-specific transcriptome analysis methods. Schematic of three different cell specific transcriptome analysis methods: FACS followed by RNA-seq, TU-tagging, and SLAM-ITseq. In a FACS-based method, cells of interest are sorted by using a marker and RNA is extracted from the sorted cells for RNA-seq analysis. Both TU-tagging and SLAM-ITseq use 4-thiouracil to label RNA by using mice expressing UPRT in the cells of interest and RNA is extracted from whole tissue containing the cells. In TU-tagging, labelled RNA is isolated by biotinylation followed by streptavidin pull-down and then RNA-seq is performed on this sorted fraction of RNA. Meanwhile, in SLAM-ITseq, alkylated total RNA is used as input for RNA-seq and bioinformatic analysis identifies labelled RNA by finding T>C containing genes.

Development • Supplementary information

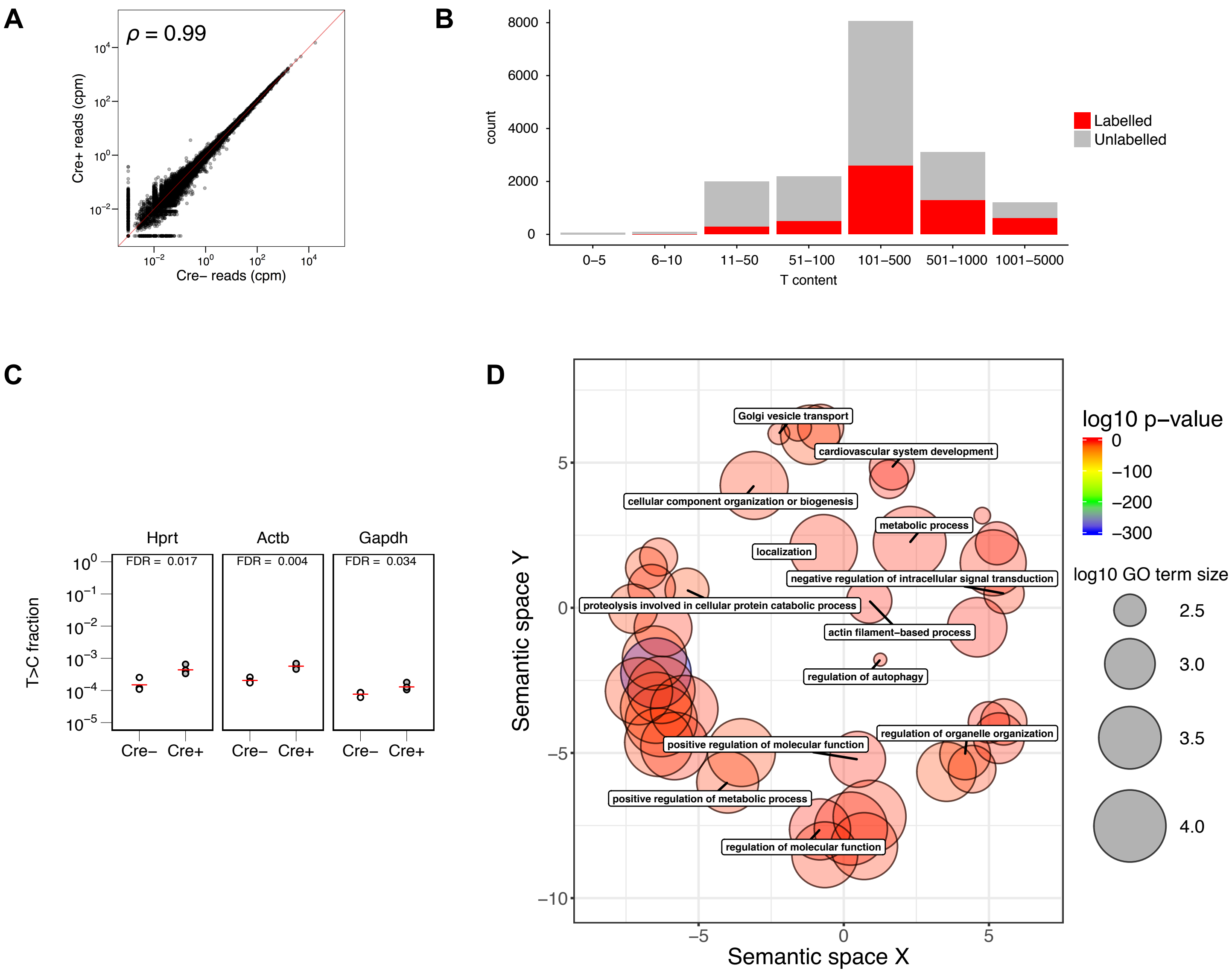

Figure S2. Additional analyses of SLAM-ITseq on endothelial cells in the brain.
(A) Comparison of RNA expression level between Cre+ and Cre- mice. Spearman's correlation coefficient ρ is shown. (B) The number of genes with different T content is shown as bar plot. Labelled and unlabelled gene count are shown as red and grey, respectively. (C) The labelling levels of the genes known to be expressed in both endothelial cells and other cells are shown. The red bars indicate the mean T>C fraction of biological replicates (Cre+: n=4, Cre-: n=3). (D) GO term enrichment analysis performed on the list of detected genes ranked by p-value in ascending order. GO terms enriched in the top of the list were further analysed by REVIGO to show clusters of similar GO terms. Representative GO terms are shown with a label.

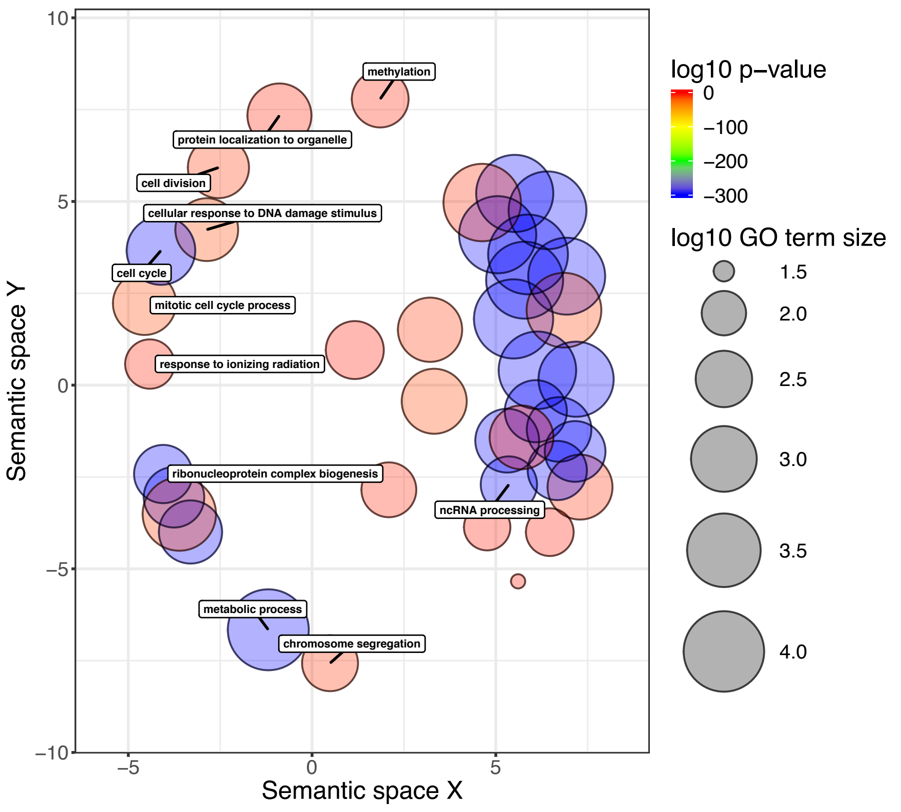

Figure S3. GO term enrichment analysis on labelled genes in intestine.
GO term enrichment analysis performed on the list of detected genes ranked by p-value in ascending order. GO terms enriched in the top of the list were further analysed by REVIGO to show clusters of similar GO terms. Representative GO terms are shown with a label.

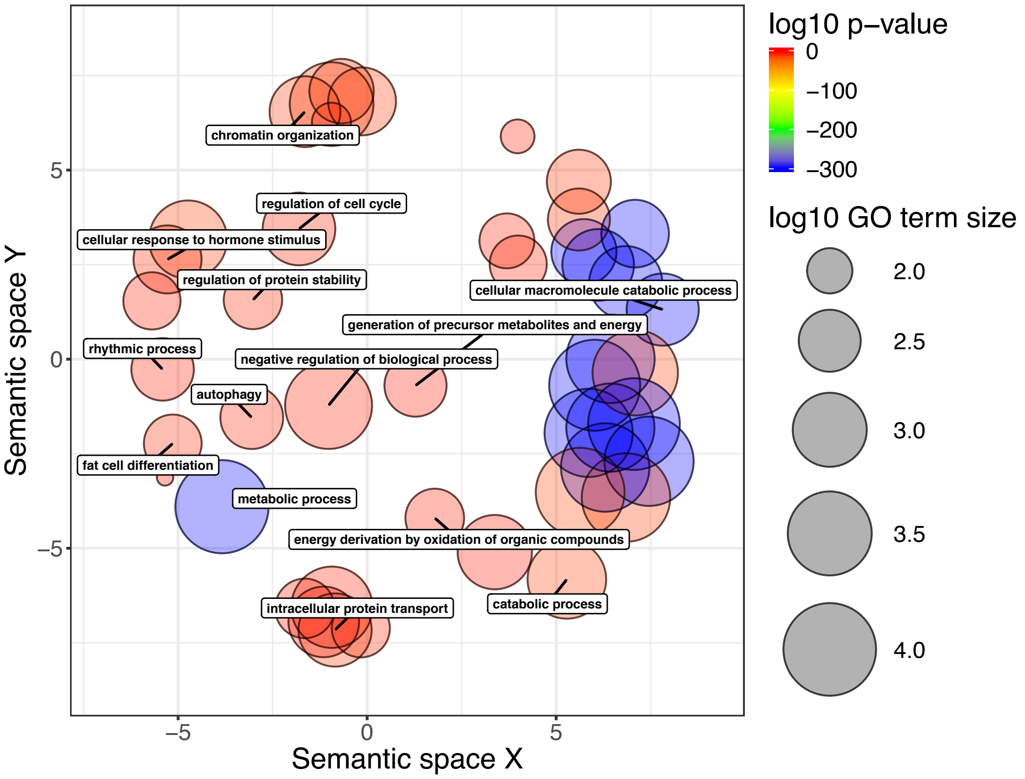

Figure S4. GO term enrichment analysis on labelled genes in eWAT.
GO term enrichment analysis performed on the list of detected genes ranked by p-value in ascending order. GO terms enriched in the top of the list were further analysed by REVIGO to show clusters of similar GO terms. Representative GO terms are shown with a label.

Supplementary tables

Table S1. T>C counts and statistical results in brain with endothelial labelling.

Click here to Download Table S1

Table S2. T>C counts and statistical results in intestine with epithelial labelling.

Click here to Download Table S2

Table S3. T>C counts and statistical results in eWAT with adipose labelling.

Click here to Download Table S3

Development • Supplementary information
